# Contextual Valence and Sociality Jointly Influence the Early and Later Stages of Neutral Face Processing

**DOI:** 10.3389/fpsyg.2016.01258

**Published:** 2016-08-19

**Authors:** Mengsi Xu, Zhiai Li, Liuting Diao, Lingxia Fan, Dong Yang

**Affiliations:** ^1^School of Psychology, Southwest UniversityChongqing, China; ^2^The School of Psychology and Cognitive Science, East China Normal UniversityShanghai, China; ^3^School of Psychology, Beijing Normal UniversityBeijing, China

**Keywords:** face processing, context effects, N2pc, EPN, LPP

## Abstract

Recent studies have demonstrated that face perception is influenced by emotional contextual information. However, because facial expressions are routinely decoded and understood during social communication, sociality should also be considered—that is, it seems necessary to explore whether emotional contextual effects are influenced by the sociality of contextual information. Furthermore, although one behavioral study has explored the effects of context on selective attention to faces, the exact underlying mechanisms remain unknown. Therefore, the current study investigated how valence and sociality of contextual information influenced the early and later stages of neutral face processing. We first employed an established affective learning procedure, wherein neutral faces were paired with verbal information that differed in valence (negative, neutral) and sociality (social, non-social), to manipulate contextual information. Then, to explore the effects of context on face perception, participants performed a face perception task, while the N170, early posterior negativity (EPN), and late positive potential (LPP) components were measured. Finally, to explore the effects of context on selective attention, participants performed a dot probe task while the N2pc was recorded. The results showed that, in the face perception task, faces paired with negative social information elicited greater EPN and LPP than did faces paired with neutral social information; no differences existed between faces paired with negative and neutral non-social information. In the dot probe task, faces paired with negative social information elicited a more negative N2pc amplitude (indicating attentional bias) than did faces paired with neutral social information; the N2pc did not differ between faces paired with negative and neutral non-social information. Together, these results suggest that contextual information influenced both face perception and selective attention, and these context effects were governed by the interaction between valence and sociality of contextual information.

## Introduction

In daily life, facial expressions are adaptive and important social communicative signals because they allow us to rapidly assess a person’s motivational state (Matsumoto et al., 2008; Graham and Labar, 2012; Xu et al., 2015). Many studies have sought to clarify the mechanism underlying face processing, most of which involved the presentation of faces in the absence of any contextual reference (Barrett et al., 2011; Wieser and Brosch, 2012). However, we usually perceive faces within certain situational contexts and rarely observe them in complete isolation. Indeed, there is growing evidence that faces do not always speak for themselves; rather, face processing is highly dependent on contextual information, such as previous experiences and information about the person represented by the face (Barrett et al., 2007, 2011; Hassin et al., 2013). Generally, contextual features can be organized into four categories: within-face features (e.g., eye gaze), within-sender features (e.g., body posture), external features from the environment surrounding the face (e.g., the visual scene), and within-perceiver features (e.g., affective learning processes; Wieser and Brosch, 2012).

Several studies have documented the modulatory effects of contextual information on face perception and reported that face perception is strongly affected by contextual information (Kim et al., 2004; Davis et al., 2010; Schwarz et al., 2013; Cloutier et al., 2014; Riggs et al., 2014). For example, Kim et al. (2004) asked participants to view surprised faces preceded by either negative or positive contextual sentences. They found that surprised faces preceded by negative contextual sentences elicited greater activation of the ventral amygdala than did those cued by positive sentences. Similarly, in a study conducted by Schwarz et al. (2013), participants viewed neutral faces preceded by contextual sentences that contained negative or positive evaluations. As predicted, contextual information modulated brain activity in response to neutral faces, with there being pronounced bilateral amygdala activity in both negative and positive conditions relative to baseline condition; while there were no differences between negative and positive context.

Further powerful evidence supporting the effects of context on face perception is provided by studies employing electroencephalography (EEG), which has the advantage of high temporal resolution (Righart and de Gelder, 2006; Morel et al., 2012; Diéguez-Risco et al., 2013; Wieser et al., 2014; Klein et al., 2015). For instance, Righart and de Gelder (2006) showed that context (fearful or neutral scenes) modulated the N170, which is particularly sensitive to face processing—that is, the amplitude of the N170 was greater for faces presented in fearful contexts than for faces in neutral contexts. In a study conducted by Wieser et al. (2014), participants viewed neutral faces preceded by sentences conveying contextual information concerning affective valence (negative, neutral, and positive) and self-reference (self-related, other-related). The findings indicated that context did not modulate the N170 component; however, modulation was observed for later components such as early posterior negativity (EPN) and the late positive potential (LPP). Specifically, faces presented in both self-related and negative contexts elicited enhanced EPN amplitude, whereas faces presented in a self-related context elicited enhanced LPP amplitude. Both of these components are associated with enhanced emotional processing and indicate relatively early (EPN) and sustained (LPP) motivated attention to salient stimuli (Hajcak et al., 2009, 2010).

In existing studies on the effects of context on face perception, most contextual information is distinguished in terms of emotionality (emotional and non-emotional) or valence (positive, negative, and neutral). However, this categorization is overly simplified, and thus consideration of at least one additional type of contextual information seems important. One such type could be sociality, or the differentiation of stimuli with social (i.e., explicitly or implicitly referencing another person) and non-social (i.e., without reference to another person) meanings (Bliss-Moreau et al., 2008). Because facial expression constitutes an important social communication signal, social information could exert a more specific or greater impact on face perception than could non-social information (Ito and Cacioppo, 2000; Hess and Hareli, 2015).

Importantly, although many studies have examined context effects in relation to face perception (Wieser and Brosch, 2012; Diéguez-Risco et al., 2013; Schwarz et al., 2013; Wieser et al., 2014), few have sought to determine whether contextual information modulates selective attention to faces. To our knowledge, only one study has addressed this topic (Anderson et al., 2011). In this study, neutral faces were initially paired with verbal information that differed in valence (negative, positive, or neutral) and sociality (social or non-social); they were then presented alone in a binocular rivalry task, where faces were presented to one eye and houses were presented to the other. The results showed that faces previously paired with negative social information were more frequently perceived in the binocular rivalry task, indicating that they had achieved attentional dominance. In contrast, no differences on dominance duration were observed between faces paired with negative and neutral non-social information. Moreover, the first percept (house or face) was not influenced at all by contextual information, which seemed to indicate that selective attention to faces (or attentional capture by faces) is not influenced by contextual information. Nevertheless, as behavioral measures are coarse (e.g., visual attention can be covertly shifted to objects and locations even without eye movements) and reflect the combined effects of a sequence of many distinct neural processes, these results should be re-examined by adopting EEG, which can provide a continuous script of neural activity and thereby illustrate how the allocation of attention unfolds over the course of a trial (Qi et al., 2013; Kappenman et al., 2014).

In summary, although existing studies have demonstrated that emotional contextual information can affect face processing, few studies have determined whether this effect is influenced by the sociality of the contextual information, let alone explored the exact underlying mechanisms (Anderson et al., 2011). Therefore, the present study aimed to address these questions. Because of its high temporal resolution, EEG was used to explore how contextual information influences the early and later stages of neutral face processing, as well as selective attention to those faces (Qi et al., 2013; Kappenman et al., 2014).

We first used an established affective learning procedure (Bliss-Moreau et al., 2008), wherein participants were presented with structurally neutral faces paired with verbal descriptions of related behavior, to manipulate contextual information in terms of valence (neutral, negative) and sociality (social, non-social). Four types of verbal description with differing contextual information were generated, including descriptions of neutral social behavior (e.g., sent messages to a friend), neutral non-social behavior (e.g., drank a glass of water), negative social behavior (e.g., hit a pregnant women), and negative non-social behavior (e.g., cut finger with a knife). Then, to explore the manner in which contextual information influenced face perception and determine whether this effect was modulated by sociality of information, participants were asked to perform a face perception task while we measured the N170, EPN, and LPP amplitudes. Finally, to explore the underlying mechanisms of context effects on selective attention to faces and re-examine whether this effect is also modulated by sociality of information, participants were instructed to perform a dot probe task while the N2pc was measured. The N2pc is a negative-going potential located at the posterior electrode sites contralateral to the location of an attended item between 150 and 320 ms subsequent to stimulus onset. As it is sensitive to the deployment of spatial selective attention, it can be used to index covert visual attention (Luck and Kappenman, 2011).

Building on previous findings and assumptions, we hypothesized that the valence and sociality of contextual information would jointly influence face perception and selective attention. Specifically, we hypothesized that faces paired with negative social information will elicit enhanced EPN and LPP amplitudes relative to faces paired with neutral social information, reflecting enhanced processing at both early and later stages. In contrast, we hypothesized that the amplitudes elicited by faces paired with negative and neutral non-social information will not differ. Regarding selective attention, we hypothesized that faces paired with negative social information will induce an attentional bias, which will manifest as greater N2pc amplitude relative to faces paired with neutral social information. Furthermore, the amplitude of the N2pc will not differ between faces paired with negative and neutral non-social information. As evidence regarding the effect of contextual information on the N170 amplitude is inconsistent, the corresponding data were analyzed using an explorative approach.

## Materials and Methods

### Participants

In total, 21 female university students aged 18–22 (*M* = 20.65, *SD* = 1.77) years participated in the study. All participants were right-handed, had normal or corrected-to-normal vision, and provided informed consent. Upon completion of the task, they received 40 RMB (approximately $6.50) for their participation. We selected only women for this experiment, because previous studies have shown sex differences in face perception (Biele and Grabowska, 2006) and word meaning comprehension (Schirmer et al., 2002). Therefore, the use of an all-female sample removed sex as a confounding variable. The data of two participants were excluded from the ERP analysis because of technical errors in the data-recording process. All experimental procedures were approved by the departmental ethics committee.

### Stimulus Selection

Sixteen greyscale photographs of different individuals with neutral expressions were selected from the Karolinska Directed Emotional Faces (Calvo and Lundqvist, 2008). We chose neutral facial expressions because they were not related to any specific emotion and were therefore susceptible to the manipulation of contextual information (Hassin et al., 2013; Schwarz et al., 2013). Faces were cropped to remove hair and resized to 135 pixels × 180 pixels.

Contextual stimuli included 16 sentences that varied in terms of valence (neutral and negative) and sociality (social and non-social), with four sentences per category. We specifically chose neutral and negative valence because the results of our pilot study indicated that it was difficult to balance arousal levels between positive and negative social sentences. In order to minimize grammatical or word-length differences between sentences, the same grammatical structure was applied throughout. Every sentence in each category contained 6–8 Chinese characters.

We performed a manipulation check by asking 20 independent judges (all women aged 18–24 years) to assess each sentence using a 9-point scale in terms of valence (1 = *negative*, 9 = *positive*) and arousal (1 = *calming*, 9 = *arousing*; **Table 1**). Two independent repeated-measures ANOVAs with contextual valence (neutral, negative) and sociality (social, non-social) as within-subjects factors were performed to separately assess valence and arousal ratings. For valence ratings, the ANOVA results revealed a significant main effect of contextual valence, *F*(1,19) = 475.80, *p* < 0.01, $\eta_{p}^{2}$ = 0.96, with more negative ratings assigned to negative sentences (*M* = 1.50, *SD* = 0.11) relative to neutral sentences (*M* = 6.21, *SD* = 0.19). Regarding the arousal ratings, the results showed a significant main effect of contextual valence, *F*(1,19) = 79.49, *p* < 0.01, $\eta_{p}^{2}$ = 0.81, with higher ratings recorded for negative sentences (*M* = 7.98, *SD* = 0.23) relative to those of neutral sentences (*M* = 5.08, *SD* = 0.24). No other significant differences were observed. Taken together, the results confirmed that these sentences were suitable for use as contextual information in the experiment.

**Table 1:** Mean affective ratings of valence and arousal (±SD) for neutral and negative sentences with social vs. non-social contexts.

	**Social**		**Non-social**	
	**Neutral**	**Negative**	**Neutral**	**Negative**
Valence	6.32 (1.00)	1.47 (0.47)	6.10 (1.00)	1.54 (0.83)
Arousal	5.10 (1.23)	7.97 (0.92)	5.07 (1.11)	7.99 (1.19)

All stimuli were displayed on a Dell computer with a 20-inch monitor. E-prime software (Psychology Software Tools, Inc., Sharpsburg, PA, USA) was used to present the stimuli and collect data.

### Design and Procedure

**FIGURE 1:**
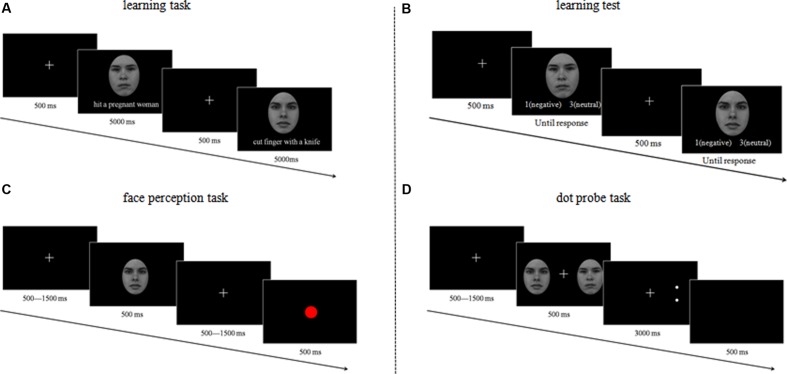
**The procedure and design used in the various tasks and phases**.

Participants were placed in an electrically shielded, soundproofed room. The experiment comprised four consecutive tasks: (a) learning task, (b) learning test, (c) face perception task; and (d) dot probe task (**Figure 1**). The first two tasks were used to manipulate contextual information and ensure the effectiveness of the manipulation, respectively. The third task was used to investigate the time course of the effect of contextual information on face perception, while the fourth task was used to explore possible attentional bias resulting from contextual information.

### Learning Task

Participants viewed the same 16 structurally neutral faces during the learning phase. Each face was paired with a sentence describing negative social (e.g., ‘hit a pregnant woman’), neutral social (e.g., ‘sent messages to a friend’), negative non-social (e.g., ‘hit finger with a hammer’), or neutral non-social (e.g., ‘drink a glass of water’) behavior, thus providing 16 different face-sentence pairs for each participant. Participants were told to imagine each target performing the behavior described in the corresponding sentence. The face–sentence pairings were counterbalanced across participants. Each face–sentence pair was displayed on the computer screen for 5 s, with a 500 ms inter-trial interval, four times in a random order.

### Learning Test

To control for potential individual differences and negative bias (i.e., negative information is learned more easily and rapidly relative to other types of information) in affective learning (Dunbar, 2004), participants completed a learning test task. This required participants to categorize the faces as neutral or negative based on the sentences that they had previously been paired with (Bliss-Moreau et al., 2008; Anderson et al., 2011). Participants were required to complete this task with a minimum of 80% accuracy before they could proceed to the face perception task. If they failed to reach the 80% threshold, they repeated the entire learning task and underwent retesting. Participants were allowed to repeat the learning task up to a maximum of five times; if they failed to reach the 80% threshold after the fifth try, they were excluded from the remainder of the experiment. In the current study, all participants exceeded the 80% threshold after completing the learning task between one and four times. On average, participants completed the learning task after 2.32 repetitions (*SD* = 0.76), with a mean accuracy of 88.89% (*SD* = 0.07). The accuracy did not differ significantly between negative (*M* = 0.89, *SD* = 0.90) and neutral sentences (*M* = 0.89, *SD* = 0.88), *t*(18) = –0.21, *p* = 0.82. In this way, we ensured that all participants had equally learned the different types of valence information before initiation of the subsequent task.

### Face Perception Task

This task began with the presentation of centrally positioned crosshairs for 500–1,500 ms, which was then followed by a neutral face or centrally positioned red dot for 500 ms. The faces in this task were grouped into four categories based on the contextual information they were paired with in the learning task: social negative face (SocNeg, for short), social neutral face (SocNeu), non-social negative face (Non-Neg), and non-social neutral face (Non-Neu). Participants viewed the face passively. To ensure that participants focused their attention on screen they were instructed to press the ‘M’ key on a keyboard as rapidly as possible after the presentation of a red dot. The task contained 200 trials (176 face and 24 red dot trials) and lasted for approximately 5 min. Each face category contained 44 trials, and each face was seen 11 times.

### Dot Probe Task

The dot probe task involved having participants determine the orientation of two small dots following the presentation of a face pair. Each trial began with the presentation of centrally positioned crosshairs for 500–1,500 ms, followed by a face pair (two different neutral faces) for 500 ms. The face pairs were classified into four categories: social negative face–social neutral face (SocNeg–SocNeu, for short), non-social negative face–non-social neutral face (Non-Neg–Non-Neu), social negative face–non-social negative face (SocNeg–Non-Neg), and social neutral face-non-social neutral face (SocNeu–Non-Neu). This manipulation allowed us to directly assess whether contextual information elicited attentional bias and whether this effect of contextual information was modulated by valence and sociality.

The face pairs were replaced by two white dots, presented on either the left- or the right-hand side of the screen. Participants were asked to press ‘1’ if the dot orientation was horizontal and ‘2’ if it was vertical. The task was designed to ensure that in half of the trials, the dots appeared in the same location as the social negative face in the SocNeg–SocNeu pair, the non-social negative face in the Non-Neg–Non-Neu pair, the social negative face in the SocNeg–Non-Neg pair, and the social neutral face in the SocNeu–Non-Neu pair; we defined these trials as the ‘congruent trials’ because we wanted to test the selective attention to faces paired with social or negative contextual information. In the other half of the trials, the dots appeared in the location opposite to that of the same faces reported in describing the congruent condition (we defined these as incongruent trials). The face and dot positions (i.e., left or right) and the target orientation (i.e., horizontal or vertical) were randomized.

In this task, reaction times (RTs) for congruent and incongruent trials were the key dependent variable. Attentional bias occurs if RTs for congruent trials are shorter than are those for incongruent trials. To ensure that eye movement artifacts did not contaminate the EEG recordings and influence the measurement of N2pc amplitude, participants were instructed to maintain visual fixation at the center of the screen throughout the trial (Kappenman et al., 2014). This task contained five blocks, each containing 96 trials, thus resulting in 480 trials in total.

### EEG Recording and Data Reduction

Electrical brain activity was recorded at 64 scalp sites, using tin electrodes mounted in an elastic cap (Brain Product, Munich, Germany), with references at the left and right mastoids and a ground electrode at the medial frontal aspect. Vertical electrooculograms (EOGs) for the right eye were recorded supra- and infraorbitally. The horizontal EOG was recorded as the left vs. the right orbital rim. EEGs and EOGs were amplified using a 0.05–100 Hz bandpass filter and continuously digitized at 500 Hz/channel. Interelectrode impedance was maintained below 5 kΩ. Offine, the data were referenced to the average of the left and right mastoids (average mastoid reference), and a bandpass filter of 0.1–30 Hz was applied. Eye movement artifacts (such as eye movements and blinking) were excluded offine. We also excluded trials with a horizontal EOG voltage exceeding ±30 μV and those contaminated with artifacts due to amplifier clipping and a peak-to-peak deflection exceeding ±80 μV. Only trials with correct responses were analyzed. Overall, for the face perception task, 1.2% of the total trials were excluded, and each face category contained about 43 trials. For the dot probe task, about 9% of the total trials were excluded and each face pair condition contained about 105 trials.

The continuous recording was divided into 700 ms epochs for each trial, beginning 200 ms prior to stimulus (i.e., face) onset (Kappenman et al., 2014). Epochs were then averaged for each participant and experimental condition. ERP components were quantified as mean amplitudes calculated over the time intervals defined in previous literature (Wieser et al., 2014; Kappenman et al., 2015; Klein et al., 2015; Zani et al., 2015). For the face perception task, the N170 (mean activity from 120 to 170 ms after stimulus onset) and EPN components (mean activity from 290 to 330 ms) were examined across two symmetrical occipital clusters of 10 electrodes around P7 and P8 (left: P5, PO5, P7, PO7, and TP7; right: P6, PO6, P8, PO8, and TP8). The LPP component (mean activity from 320 to 420 ms after stimulus onset) was analyzed as an index of sustained motivated attention across a frontocentral cluster of 10 electrodes (F1, F2, F3, F4, Fz, FC1, FC2, FC3, FC4, and FCz; Klein et al., 2015). For the dot probe task, the measurement of the N2pc component (mean activity from 268 to 308 ms after face stimulus onset) focused on PO7 and PO8, where the N2pc has been found to be maximal (Yao et al., 2014).

### Data Analysis

#### Behavioral Analysis

The accuracy of responses to the red dot was measured in the face perception task. In the dot probe task, accuracy and RTs (for correct response trials only) were analyzed separately using a within-participants ANOVA with congruence (congruent, incongruent) and face pair (SocNeg–SocNeu, Non-Neg–Non-Neu, SocNeg–Non-Neg, and SocNeu–Non-Neu) as factors.

### ERP Analysis

First, we investigated the time course of the effects of contextual information on face perception. For the face perception task, repeated-measures ANOVAs with contextual valence (neutral, negative) and sociality (social, non-social) as factors were performed to assess differences in N170, EPN, and LPP amplitudes.

Second, we attempted to explore the possible attentional bias resulting from contextual information. For the dot probe task, repeated-measures ANOVAs with contralaterality (contralateral, ipsilateral) and face pair (SocNeg–SocNeu, Non-Neg–Non-Neu, SocNeg–Non-Neg, and SocNeu–Non-Neu) as factors were performed to assess differences in N2pc amplitudes. The contralateral waveform was calculated as the average of the left- and right-sided electrodes for the right- and left-sided targets, respectively (thus taking into account activity elicited by the target). In contrast, the ipsilateral waveform was calculated as the average of the left- and right-sided electrodes for the left- and right-sided targets, respectively (thus taking into account activity elicited by the non-target; Kappenman et al., 2015). Targets in the dot probe task were defined as the social negative face in the SocNeg–SocNeu pair, the non-social negative face in the Non-Neg–Non-Neu pair, the social negative face in the SocNeg–Non-Neg pair, and the social neutral face in the SocNeu–Non-Neu pair.

## Results

### Behavioral Data

In the face perception task, the mean accuracy for responses to the red dot was above 97% (*M* = 97.11%, *SD* = 0.06), suggesting that participants focused their attention on the task.

In the dot probe task, the mean accuracy was also above 97%, and the main effects of congruency and face pair and the interaction between them were non-significant, *p*s > 0.44. For the analyses of RTs, the main effects of congruency and face pair and the interaction between them were non-significant, *p*s > 0.40.

### ERP Data

#### The Effect of Contextual Information on Face Perception

**FIGURE 2:**
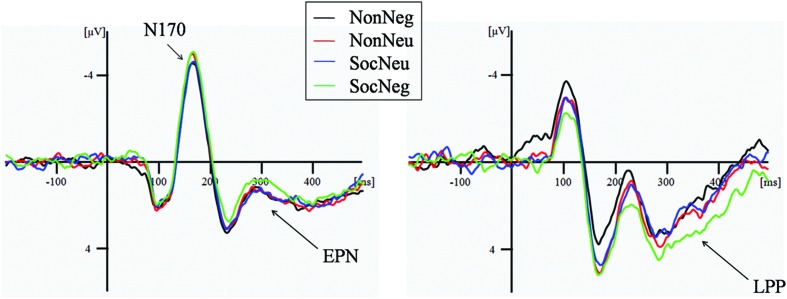
**Illustration of the N170, EPN, and LPP components for each condition during the face perception task (N170 and EPN: average of P5, P6, PO5, PO6, P7, P8, PO7, PO8, TP7, and TP8; LPP: average of F1, F2, F3, F4, Fz, FC1, FC2, FC3, FC4, and FCz)**. Non-Neg, non-social negative face; Non-Neu, non-social neutral face; SocNeg, social negative face; SocNeu, social neutral face; EPN, early posterior negativity; LPP, late posterior potential.

In the face perception task, the ANOVA conducted to assess differences in N170 amplitude yielded no significant results; in other words, the main effects of contextual valence and sociality and the interaction between them were non-significant (negative social face: *M* = –1.38 μV, *SD* = 2.21; neutral social face: *M* = –0.90 μV, *SD* = 2.23; negative non-social face: *M* = –1.00 μV, *SD* = 2.25; neutral non-social face: *M* = –1.10 μV, *SD* = 2.49), *p*s > 0.14 (**Table 2**; **Figure 2**).

**Table 2:** Mean (±SD) N170, EPN, and LPP amplitudes for each experimental condition in the face perception task.

	**SocNeg**	**SocNeu**	**Non-Neg**	**Non-Neu**
N170	−1.38 (2.21)	−0.90 (2.23)	−1.00 (2.25)	−1.10 (2.49)
EPN	1.29 (2.17)	1.89 (2.15)	2.02 (1.98)	1.91 (2.19)
LPP	2.92 (3.00)	1.88 (1.96)	1.75 (2.48)	2.07 (2.70)

*EPN, early posterior negativity; LPP, late posterior potential; Non-Neg, non-social negative face; Non-Neu, non-social neutral face; SocNeg, social negative face; SocNeu, social neutral face.*

With respect to EPN amplitude, the ANOVA results revealed a significant main effect of sociality, *F*(1,18) = 5.29, *p* = 0.03, $\eta_{p}^{2}$ = 0.22: social faces (*M* = 1.59 μV, *SD* = 2.05) elicited a more negative EPN amplitude than did non-social faces (*M* = 1.97 μV, *SD* = 2.02). Moreover, the interaction between contextual valence and sociality was significant, *F*(1,18) = 4.45, *p* = 0.04, $\eta_{p}^{2}$ = 0.20. Further analyses indicated that negative social faces (*M* = 1.29 μV, *SD* = 2.17) elicited a marginally more negative EPN amplitude than did neutral social faces (*M* = 1.89 μV, *SD* = 2.15), *F*(1,18) = 3.89, *p* = 0.06, $\eta_{p}^{2}$ = 0.18. In contrast, the EPN amplitude did not differ between negative (*M* = 2.01 μV, *SD* = 1.98) and neutral (*M* = 1.91 μV, *SD* = 2.19) non-social faces, *F*(1,18) = 0.19, *p* = 0.67, $\eta_{p}^{2}$ = 0.01 (**Table 2**; **Figure 2**).

With respect to LPP amplitude, the ANOVA results revealed a significant main effect of sociality, *F*(1,18) = 4.83, *p* = 0.04, $\eta_{p}^{2}$ = 0.21, which indicated that social faces (*M* = 2.40 μV, *SD* = 2.36) elicited greater LPP amplitudes than did non-social faces (*M* = 1.91 μV, *SD* = 2.34). Moreover, the interaction between contextual valence and sociality was significant, *F*(1,18) = 4.86, *p* = 0.04, $\eta_{p}^{2}$ = 0.21. Further analyses showed that negative social faces (*M* = 2.92 μV, *SD* = 3.00) elicited a greater LPP amplitude than did neutral social faces (*M* = 1.88 μV, *SD* = 1.96), *F*(1,18) = 6.10, *p* = 0.02, $\eta_{p}^{2}$ = 0.25. In contrast, the LPP amplitude did not differ between negative (*M* = 1.75 μV, *SD* = 2.48) and neutral (*M* = 2.07 μV, *SD* = 2.70) non-social faces, *F*(1,18) = 0.38, *p* = 0.54, $\eta_{p}^{2}$ = 0.02 (**Table 2**; **Figure 2**).

#### The Effect of Contextual Information on Attentional Bias

**FIGURE 3:**
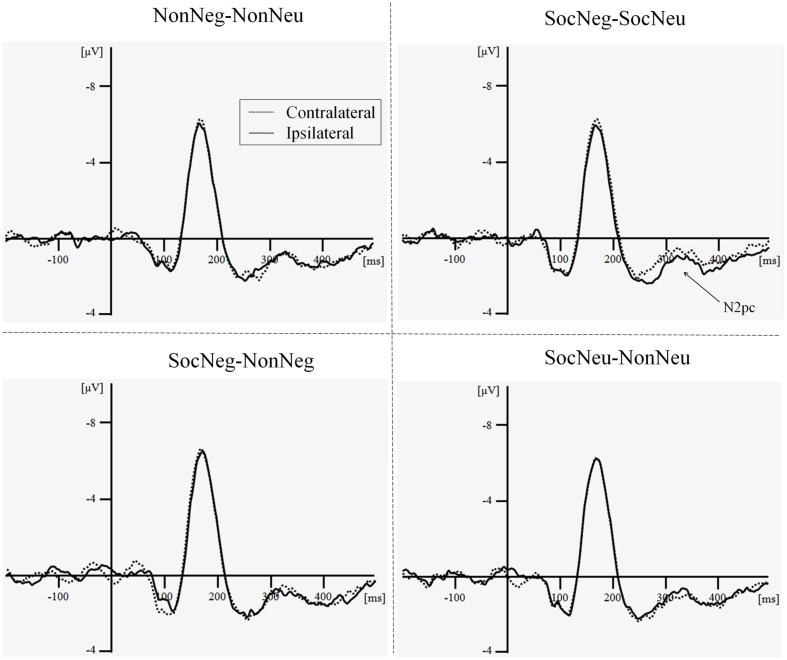
**Illustration of the N2pc component for each condition during the dot probe task (average of PO7 and PO8)**. Non-Neg–Non-Neu, non-social negative face–non-social neutral face; SocNeg–SocNeu, social negative face–social neutral face; SocNeg–Non-Neg, social negative face–non-social negative face; SocNeu–Non-Neu, social neutral face–non-social neutral face.

In the dot probe task, the results of the ANOVA conducted to examine differences in N2pc amplitude showed a significant interaction between contralaterality and face pair, *F*(3,16) = 4.59, *p* = 0.03, $\eta_{p}^{2}$ = 0.42. Further analysis indicated that, for the SocNeg–SocNeu pair, the contralateral condition (*M* = 1.15 μV, *SD* = 2.96) evoked a more negative N2pc amplitude than did the ipsilateral condition (*M* = 1.80 μV, *SD* = 3.11), *F*(1,18) = 13.20, *p* < 0.01, $\eta_{p}^{2}$ = 0.42. This suggests the existence of an attentional bias to faces paired with negative, compared to neutral, social information (**Table 3**; **Figure 3**). For other face pairs, no significant results were observed, *p*s > 0.20.

**Table 3:** Mean (±SD) accuracy, response times (RTs), and N2pc amplitudes for each experimental condition in the dot probe task.

	**Non-Neg–Non-Neu**	**SocNeg–SocNeu**	**SocNeg–Non-Neg**	**SocNeu–NonNeu**
**Accuracy**				
Contralateral	0.98 (0.02)	0.98 (0.01)	0.98 (0.02)	0.98 (0.02)
Ipsilateral	0.98 (0.02)	0.98 (0.02)	0.98 (0.02)	0.98 (0.02)
**RTs**				
Contralateral	573.27 (74.14)	569.30 (80.87)	571.40 (78.19)	567.30 (78.72)
Ipsilateral	575.81 (80.34)	570.67 (81.07)	567.43 (78.85)	569.91 (81.51)
**N2pc**				
Contralateral	1.66 (3.06)	1.15 (2.96)	1.41 (2.69)	1.63 (2.86)
Ipsilateral	1.41 (2.81)	1.80 (3.11)	1.41 (2.63)	1.44 (2.88)

*Non-Neg–Non-Neu, non-social negative face–non-social neutral face; SocNeg–SocNeu, social negative face–social neutral face; SocNeg–Non-Neg, social negative face–non-social negative face; SocNeu–Non-Neu, social neutral face–non-social neutral face.*

## Discussion

The aims of the present study were to determine the following: (1) the manner in which contextual information influences face perception, and especially the role of sociality of contextual information; and (2) the mechanisms underlying the effects of contextual information on selective attention to neutral faces. To this end, neutral faces were initially paired with verbal information that differed in valence (negative, neutral) and sociality (social, non-social); they were then presented in face perception and dot probe tasks while ERPs were concurrently measured. Consistent with our hypotheses on face perception, faces paired with negative social information elicited greater EPN and LPP amplitudes than did faces paired with neutral social information. In contrast, the EPN and LPP amplitudes did not differ significantly between faces paired with negative and neutral non-social information. Furthermore, consistent with our hypotheses on selective attention, faces paired with negative social information elicited a more negative N2pc amplitude (indicating an attentional bias) than did faces paired with neutral social information, while the N2pc amplitude did not differ between faces paired with negative and neutral non-social information.

Many previous studies have explored the effects of context on face processing. Typically, they have found that emotional contextual information can affect face processing (Anderson et al., 2011; Wieser and Brosch, 2012; Schwarz et al., 2013; Riggs et al., 2014; Wieser et al., 2014). However, few studies have looked at both sociality (social, non-social) and valence as contextual factors (Anderson et al., 2011). Because facial expression is an important social signal, we hypothesized that social information would exert a specific impact on face processing. This led to our manipulation of both valence and sociality in the present study. Moreover, our method of manipulation can be considered somewhat more ecologically valid than past methods. Unlike the majority of previous studies (but see Anderson et al., 2011 for a similar method), wherein contextual information was presented only a few seconds before the target faces, thus leading to relatively brief context effects, our affective learning procedure had participants learn the contextual information first. Only after robust learning of contextual information did participants complete the face processing tasks, which is more reflective of daily life because we usually evaluate other people based on previous experiences.

Using this manipulation method, we initially replicated the effects of context on face perception reported in previous studies (Wieser et al., 2014; Klein et al., 2015). Specifically, we found that faces paired with negative social information elicited greater EPN and LPP amplitudes than did faces paired with neutral social information. Because the EPN and LPP components are associated with enhanced emotional processing and indicate relatively early (EPN) and sustained (LPP) motivated attention to salient stimuli (Hajcak et al., 2009, 2010), the observed EPN and LPP amplitude changes suggest that the negative social faces had in fact acquired the emotional valence of the context and underwent faster and longer-lasting processing relative to neutral social faces. These results were understandable because negative social stimuli attract greater attention and receive preferential processing compared to neutral social stimuli; this is likely because they are potentially threatening (Olofsson et al., 2008) and violate social norms.

Moreover, the EPN and LPP amplitudes did not differ between the faces paired with negative and neutral non-social information. These results were interesting and have not been reported previously. Because neither the valence nor the arousal ratings for the contextual sentences indicated a difference between social and non-social information, the current absence of differences in these ERP amplitudes could suggest that the context effect was specifically related to *negative social* information rather than arousing or negative stimuli in general. These results can be explained by the social nature of facial expressions (Wieser and Brosch, 2012; Hess and Hareli, 2015). More specifically, as facial expressions are routinely decoded and understood during social communication, they are strongly influenced by social contextual information. Taken together, the results of the analysis of EPN and LPP amplitudes confirmed the effects of contextual information on face perception, and importantly, indicated that these effects were influenced by the interaction between valence and sociality of contextual information.

Nevertheless, no significant results were observed for the N170 component, which was inconsistent with Righart and de Gelder (2006). Because the N170 is a component related to the structural encoding of faces, it would be only modulated by physical features (e.g., absence or presence of certain key features, face inversion) and by emotional expressions which modify the physical configuration of the face (Eimer, 2000; Luo et al., 2010). Consequently, this discrepancy between current study and Righart and de Gelder (2006) might be related to the method of context manipulation. To be specific, Righart and de Gelder (2006) superimposed the faces on the context in a display, thus the context might have disrupted encoding of the faces and thereby influenced the N170 component. While in current study, the faces were presented alone after the contextual manipulation, and in any case, contextual top down information would not alter the physical features of the face by definition, and in consequence they would not impact the N170.

Our second aim was to explore the effects of context on selective attention to faces. At the behavioral level, no significant results were observed to support the attentional bias hypothesis. Nevertheless, in measuring the ERPs, we observed significant differences in N2pc amplitudes between negative and neutral social faces. This discrepancy between behavioral and ERP data (i.e., the fact that the attentional bias existed only in the ERP data) is usually observed in the dot probe task and has been explored in recent studies (Kappenman et al., 2014, 2015). Kappenman et al. (2014) examined this issue and suggested that the absence of this result for behavioral data was caused by limitations of behavioral measures: namely, they only reflect the combined effects of a sequence of many distinct neural processes. In contrast, ERPs could provide a continuous script of neural activity and therefore could show how the allocation of attention unfolds over the course of a trial. In light of this, behavioral results from the dot probe task could be unreliable. Future researchers should consider this possibility.

At the neural level, we found that faces paired with negative social information elicited greater N2pc amplitude relative to that of faces paired with neutral social information, suggesting the existence of attentional bias toward faces in negative social contexts. These results were partly consistent with those of Anderson et al. (2011), who found that faces within a negative emotional context gained attentional dominance. However, our results further demonstrated that negative social context biased attention toward neutral faces at a very early stage, which was inconsistent with Anderson et al. (2011; i.e., ‘the first percept seen’ in Anderson et al., 2011 was not influenced by contextual information). This discrepancy could be explained by the fact that ERPs provide a more fine grained measure of attentional deploying and could thus be more efficacious in assessing attentional capture, compared to behavioral measures (Qi et al., 2013; Kappenman et al., 2014). Furthermore, the attentional bias toward faces paired with negative social information is reasonable: previous studies have demonstrated that the perception and evaluation of faces is substantially influenced by contextual information such as that obtained second-hand (i.e., descriptive affective sentences; Wieser and Brosch, 2012; Schwarz et al., 2013; Wieser et al., 2014). As a result, hearing that a person stole, lied, or cheated could cause the perceiver to treat structurally neutral but purportedly villainous faces as threatening, thus causing them to deploy more attentional resources to ensure the rapid detection of such faces. It is easy to imagine that this advantage in detecting ‘bad people’ could protect us from potential danger.

No attentional bias was observed for faces paired with negative or neutral non-social information. This suggests that the differences in attentional bias toward faces resulting from contextual information specifically related to negative social information rather than arousing or negative stimuli in general. Taken together, these results confirmed the effects of contextual information on selective attention, and, importantly, indicated that these effects were influenced by the interaction between valence and sociality. In this way, the findings are rather intriguing and novel. While the evidence to date has provided us with an understanding of the manner in which contextual information influences face perception, the current study extended these contextual effects to the attentional processing of faces, and for the first time, directly demonstrated that negative social contextual information can elicit attentional bias.

This study has several limitations. First, we recruited only women as participants in order to exclude sex as a confounding variable (Biele and Grabowska, 2006). Therefore, our results cannot be generalized to men. Future studies should provide a comparison between the sexes in order to determine whether the findings are generalizable to both. Second, the potential influence of individual differences was not considered (Lee et al., 2012). Some studies have shown that social anxiety influences individuals’ perception and evaluation of social stimuli (Schwarz et al., 2013); therefore, future studies should consider this issue and determine whether individual differences influence the effects of contextual information on face processing. Third, in the learning test, we asked participants to categorize the faces as neutral or negative, but did not ask them to assess whether the action associated to the faces were social (targetting others) or non-social (targetting themselves). Consequently, we cannot be sure that there were no differences in the affective learning of social and non-social information. Therefore, future studies should examine this issue. Another question in the learning task and test was that we failed to control for the working memory ability of participants. This should be considered in further studies because the affective learning procedure used here might be related to working memory ability (Fan et al., 2016). Fourth, although we endeavored to divide the contextual information into social and non-social sentences, we could not be certain that the non-social sentences did not elicit any social emotion. For example, participants could exhibit empathy when imagining someone being bitten by a snake. Further research is therefore required to explore new methods of distinguishing between non-social and social information. Finally, the study focused on the effects of contextual information on the processing of neutral faces. As affective faces are equally common in daily life, an interesting next step would be to explore the effects of context on the processing of affective faces. In addition, our current study failed to include positive contextual information, which should be looked at in the future.

## Conclusion

The present study examined the effects of context on face perception and selective attention. The results showed that contextual information provided by verbal description could modulate face perception and selective attention in an entirely top-down manner, independent of basic structural facial features. More importantly, these context effects were influenced by the interaction between valence and sociality of contextual information.

## Author Contributions

Conceived and designed the experiments: MX, ZL, and DY. Performed the experiments: MX, ZL, LD, and LF. Analyzed the data: MX, ZL, LF, and DY. Contributed reagents/materials/analysis tools: MX, ZL, LD, LF, and DY. Wrote the paper: MX, ZL, LD, LF, and DY.

## Conflict of Interest Statement

The authors declare that the research was conducted in the absence of any commercial or financial relationships that could be construed as a potential conflict of interest.
